# Distinct cell division features in *Anabaena*, a multicellular cyanobacterium

**DOI:** 10.1128/jb.00032-25

**Published:** 2025-06-17

**Authors:** Antonia Herrero

**Affiliations:** 1 Instituto de Bioquímica Vegetal y Fotosíntesiss, CSIC and Universidad de Sevilla, Seville, Spain; Queen Mary University of London, London, United Kingdom

**Keywords:** filamentous cyanobacteria, heterocyst differentiation, intercellular septa, peptidoglycan growth, Z-ring

## Abstract

Filamentous cyanobacteria such as *Anabaena* display a distinct multicellular organization in the bacterial kingdom. In the filament, the cells share a common periplasm and continuous peptidoglycan sacculus and outer membrane. This structure is propagated by cell division proteins with specific features, including the Z-ring components FtsZ, ZipN, and SepF. Septal junction protein complexes, which provide cell-cell cohesion and communication functions key to the *Anabaena* multicellular behavior, are recruited to the intercellular septa by interaction with ZipN and SepF during cell division. *Anabaena* also shows specific features in relation to peptidoglycan growth. The activities of the elongasome and the divisome complexes are interdependent, and septal growth is maintained throughout the cell cycle, contributing to the determination of the proper filament geometry and building and maintaining intercellular communication structures. During the differentiation of heterocysts, cells specialized for the fixation of atmospheric nitrogen, cell division is lost, setting the point of commitment to differentiation. Genes encoding Z-ring components are repressed, and the incorporation of these components into functional divisional complexes is inhibited, involving specific regulatory proteins in connection to specificities of the *Anabaena* Z-ring. The essential dependence of FtsZ polymerization on SepF provides a mechanism for Z-ring inhibition by downregulating SepF during differentiation.

## INTRODUCTION

Cyanobacteria are oxygenic phototrophs that make an important contribution to the global primary productivity in the biosphere (1). Indeed, they are the organisms in which oxygenic photosynthesis, based on the operation of two serial photosystems, evolved, eventually leading to the accumulation of oxygen in the Earth’s atmosphere. From then on, this event conditioned the evolution of life on our planet (2). Furthermore, cyanobacteria represent the phylogenetic ancestors of the chloroplast, the photosynthetic organelle of algae and plants (3). In addition to the key role of cyanobacteria in the global carbon cycle, many cyanobacteria carry out the fixation of atmospheric nitrogen, being either in free-living or symbiotic form, major N_2_-fixers in the Earth’s oceans (4).

**Fig 1:**
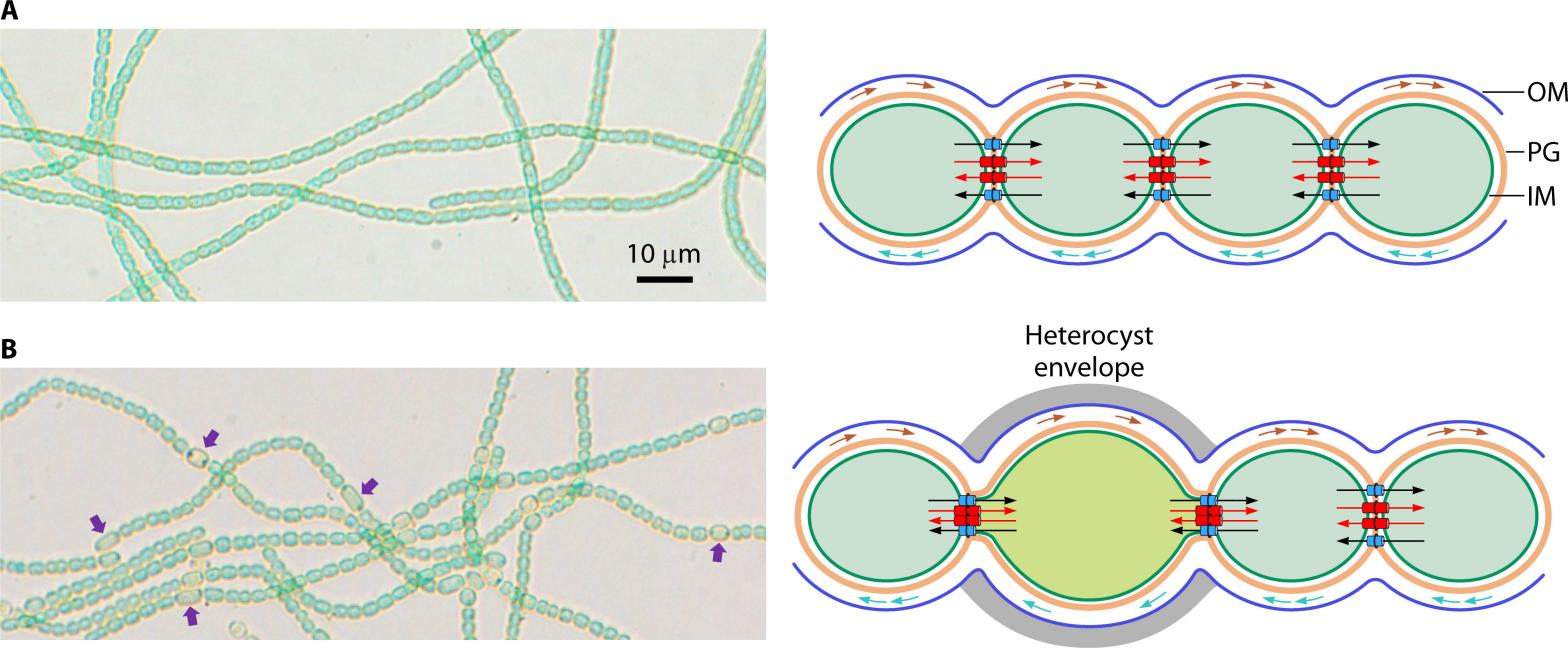
Images of filaments of *Anabaena* growing with nitrate (upper part) or diazotrophically (lower part). Some heterocysts in the latter are indicated with purple arrows. Magnification is the same for both micrographs. A schematic of the filament structure is presented to the right. OM, outer membrane; PG, peptidoglycan; IM, cytoplasmic membrane. *Septal junction* complexes are represented in the intercellular regions. Periplasmic and septal arrows indicate molecular transfer.

Many cyanobacteria grow forming filaments that display traits of multicellularity, a remarkable biological innovation that emerged early in the evolution of the phylum and has contributed importantly to cyanobacterial diversification and abundance (2, 5–7). Organisms of the order Nostocales form filaments of communicated cells that, depending on the environmental conditions, can include different cell types specialized in various biological tasks (8–10). Regarding the structure of the filament, cyanobacteria are diderm bacteria classified into the Terrabacteria (11). However, whereas each cell is enclosed by an individual cytoplasmic membrane, the outer membrane is continuous along the filament, delimiting a continuous periplasm that harbors a continuous peptidoglycan sacculus that surrounds each cell and is engrossed in the intercellular regions. In addition, multiprotein complexes, termed *septal junctions*, expand the septal regions between consecutive cells, providing channels that connect their cytoplasm (Fig. 1).

### Heterocyst differentiation

One type of differentiated cell occurring in many filamentous cyanobacteria is the heterocyst, which is specialized for the fixation of atmospheric nitrogen in oxic environments. Thus, in the presence of combined nitrogen (e.g., ammonium or nitrate), all the cells in the filament are equivalent and perform oxygenic photosynthesis. However, under conditions of combined nitrogen limitation (diazotrophic growth), heterocysts are formed from certain vegetative cells of the filament, and they display multiple morphological, structural, and physiological differences with regard to the non-differentiated cells of the filament (Fig. 1). Specific features of the heterocysts include the presence of additional cell-envelope glycolipid and polysaccharide layers that limit O_2_ penetration into the cytoplasm; narrowing of the cell-cell contacts at the intercellular septa; inactivation of O_2_-evolving Photosystem II; expression of the N_2_-fixation machinery including nitrogenase; and alteration of pathways for assimilation of ammonium (in heterocysts resulting from N_2_ reduction) (8–10). These differences arise from a complex developmental program that includes major events of regulation of gene expression, which are orchestrated mainly by two transcriptional regulators, NtcA and HetR, that are essential for differentiation (see Reference 12). NtcA is a cyanobacterial-specific CRP-type transcriptional regulator that coordinates responses to carbon and nitrogen availability, including heterocyst differentiation, with a global impact on gene expression. HetR is specific for filamentous cyanobacteria and exhibits a DNA-binding activity that is required for activation of expression of multiple genes involved in differentiation. Heterocyst differentiation is irreversible beyond a certain stage, and commitment to differentiation has been related to the loss of cell division capacity of the differentiating cells involving a multifactorial regulatory network (see Reference 10).

### Intercellular communication

In the differentiated diazotrophic filament, a division of labor operates between N_2_-fixing heterocysts and CO_2_-fixing vegetative cells. Heterocysts transfer products of the N_2_ fixation process, mainly in the form of amino acids (including a dipeptide), to vegetative cells. Meanwhile, vegetative cells transfer reduced carbon compounds to heterocysts, which use them as sources of reductants and energy and as carbon skeletons for the incorporation of the ammonium resulting from N_2_ reduction (reviewed in Reference 9). Besides this nutritional exchange, a traffic of morphogens (e.g., PatS- and HetN-related peptides) that establish the spatial pattern of heterocyst distribution takes place between heterocysts and vegetative cells. In *Anabaena*, heterocysts are found separated by stretches of ca. 10 vegetative cells along the filament and can also occur at the filament ends (8).

**Fig 2:**
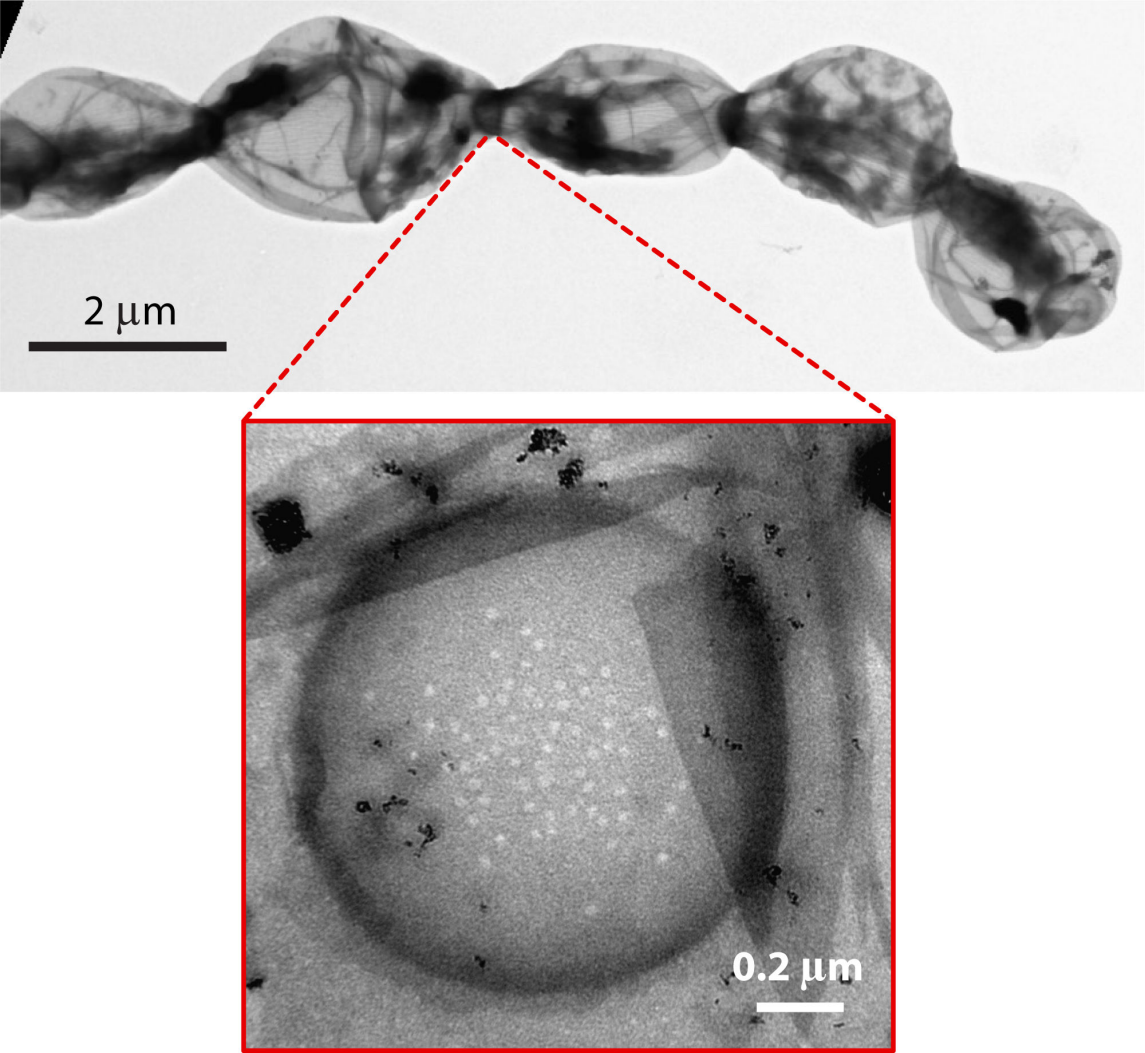
Electron micrograph of isolated murein sacculus from a fragment of an *Anabaena* filament grown with ammonium. Note the engrossed septal disks in the intercellular spaces. The lower part of the figure shows a septal disk displaying the nanopore array.

The shared periplasm could be considered a communication conduit along the filament (13). In addition, septal junctions have been extensively studied and shown to represent paths for intercellular molecular exchange (14, 15). Septal junction complexes traverse the septal peptidoglycan disk through nanopores, which in *Anabaena* are found in arrays of ca. 50 nanopores per disk on average, with a tendency to concentrate in its central part (16) (Fig. 2). Generally, septal junction components are polytopic proteins including coiled-coil motifs. For example, SepJ, a key septal protein, is a polytopic component with multiple transmembrane helices and a long coiled-coil domain (9), and FraC and FraD are also membrane proteins that have been specifically localized to some septal junctions (15). Some of these proteins might be localized to septal junction structures by interaction with the integral membrane components, such as in the case of the SepJ-interacting protein SepI (17). Inactivation or deletion of septal junction component-encoding genes frequently impairs the structure of the septal nanopore array and/or the activity of intercellular molecular transfer. In addition, the loss of SepJ, FraC, FraD, or SepI leads to a phenotype of filament fragmentation, indicating that these proteins contribute to the filament cohesion (see References 14, 17–19).

This distinct bacterial multicellular organization is propagated by a cell division process that contrasts with the cell division process that produces separate siblings in unicellular bacteria. In this review, I deal with specific features of cell division in the model multicellular heterocyst-forming cyanobacterium *Anabaena* sp. strain PCC 7120 (hereafter *Anabaena*), as well as with the interplay between peptidoglycan expansion during cell growth and cell division, and between inhibition of cell division and heterocyst differentiation.

## SPECIFIC FEATURES OF CELL DIVISION IN *ANABAENA*

### The *Anabaena* Z-ring

In the vast majority of bacteria, cell division is initiated by the polymerization of the tubulin homolog FtsZ. FtsZ is connected to the inner surface of the cytoplasmic membrane by interaction with different adaptor proteins that have membrane-interacting domains, the so-called FtsZ-tethers, which also organize the FtsZ polymers to form a dynamic internal ring (the Z-ring), normally positioned at midcell (20). The Z-ring serves as a scaffold for the recruitment of multiple other proteins, some of which have periplasmic parts, to constitute the multiprotein complex termed the divisome. This complex includes the enzymes for peptidoglycan processing to synthesize the new poles of the daughter cells and finally directs envelope constriction and daughter cell separation. The *Anabaena* genome contains genes encoding homologs of some of the divisome components of model bacteria, including FtsQ, FtsK, FtsW, FtsI, FtsE, and FtsX (see Reference 21), although, in general, their role in *Anabaena* has been scarcely studied (see Reference 22).

**Fig 3:**
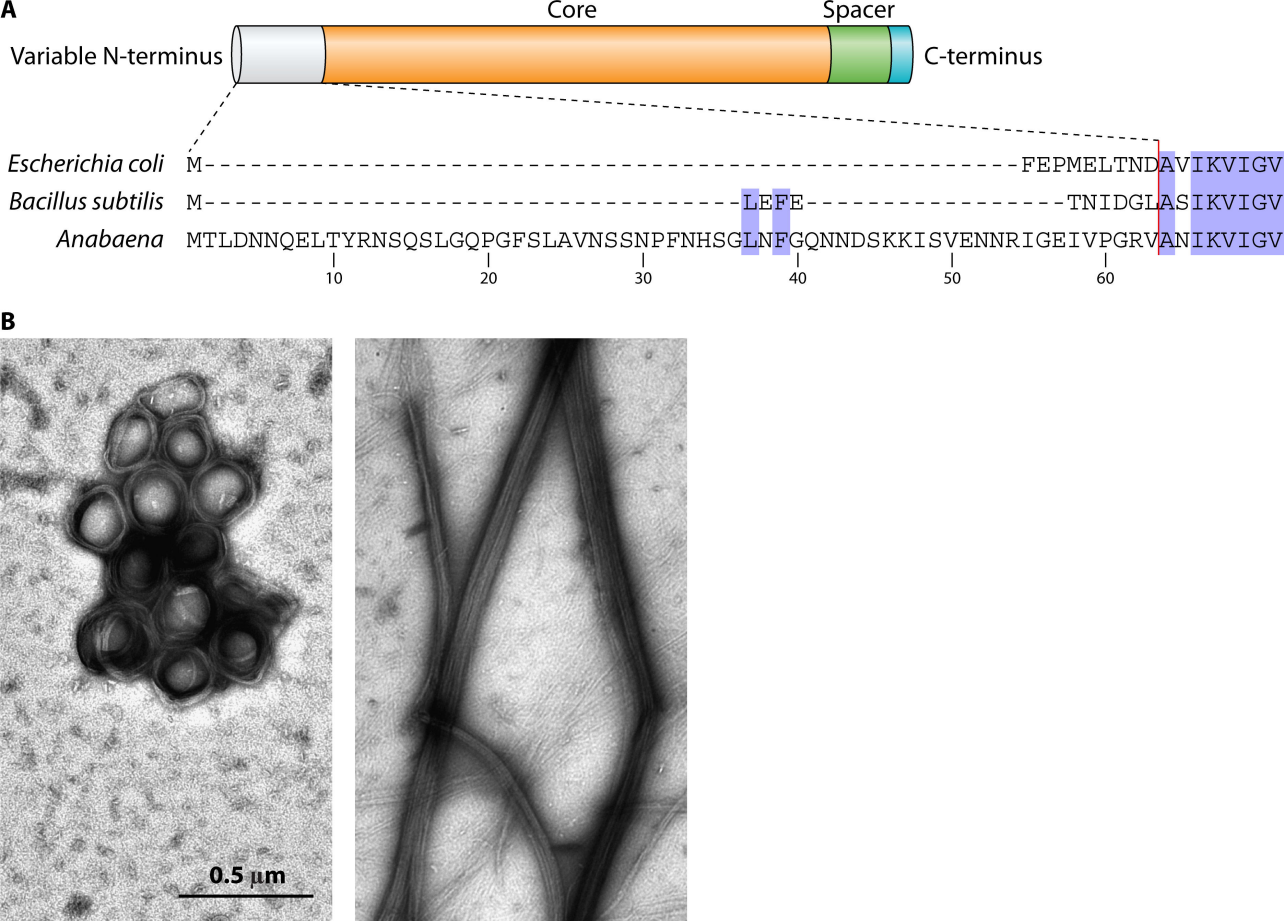
(**A**) Schematic of the general regions of bacterial FtsZ and the sequence of the N-terminus from the indicated organisms. (**B**) Polymers of *Anabaena* FtsZ and ΔN-FtsZ (lacking residues 2–51) formed *in vitro* and visualized by transmission electron microscopy after negative staining with uranyl acetate (see Reference 24). Magnification is the same for both micrographs.

Regarding components of the Z-ring, FtsZ of *Anabaena* presents the generally conserved globular core that includes the determinants for GTP binding and hydrolysis, the variable unstructured spacer, and the C-terminal conserved peptide, which includes the sites of interaction of the most common FtsZ partners (see Reference 23). In addition to these domains, *Anabaena* FtsZ contains an N-terminal peptide of ca. 60 amino acid residues that precedes the globular core and is specific and highly conserved in the clade of filamentous cyanobacteria capable of cell differentiation (24) (Fig. 3A). This peptide is essential for viability, and even strains that express an FtsZ variant lacking this part (ΔN-FtsZ) together with the native FtsZ display giant aberrant cells and asymmetric septation, indicative of a drastic impairment in cell division, under conditions of prevalence of the truncated protein (24). *In vitro*, FtsZ from *Anabaena* polymerizes forming thick toroidal bundles (Fig. 3B) that differ from the single protofilaments generally formed by FtsZ, for example, from *Escherichia coli*, when incubated under similar conditions (25). The toroidal bundles formed by the native *Anabaena* FtsZ also differ from the rigid linear bundles formed by ΔN-FtsZ (24) (Fig. 3B). It is possible that the N-terminal peptide affects the curvature and thickness of *Anabaena* FtsZ polymers by influencing the tendency for longitudinal and/or lateral interactions of FtsZ subunits.

Cyanobacteria, in general, also present some specific cell division proteins, such as ZipN (a.k.a. Ftn2) and ZipS (a.k.a. Ftn6) (26). ZipN encompasses cytoplasmic and periplasmic domains separated by a transmembrane helix. In *Anabaena*, ZipN is an essential component of the divisome and a principal membrane tether of FtsZ, which remains soluble in the cytoplasm to a high extent upon downregulation of the *zipN* gene, a situation that also leads to the formation of aberrant giant cells prone to cell lysis (27). Although having a Gram-negative-type cell envelope, cyanobacteria, in general, possess homologs of SepF, a protein characteristic of Gram-positive bacteria in which SepF represents a well-identified, generally dispensable, FtsZ tether (e.g., 28); nonetheless, in Actinobacteria, which lack the FtsZ tethers FtsA and ZipA, SepF is essential (29). In *Anabaena*, SepF is essential, and whereas strains that down-express *sepF* are non-viable, strains overexpressing it exhibit drastically elongated cells indicative of cell division defects, pointing to a requirement of very precise SepF levels for effective cell division (30). Thus, both ZipN and SepF appear to have functions beyond the localization of FtsZ to the cytoplasmic membrane. Indeed, FtsA negatively regulates FtsZ bundling, influencing its polymerization dynamics (31). It has been discussed that in *E. coli* and *BacillusΔ subtilis*, FtsA and SepF, respectively, may have a role in the stabilization of the division ring (20). In the case of *Anabaena* ZipN, it can interact (according to Bacterial Adenylate Cyclase Two-Hybrid, BACTH, assays) with most of the known components of the divisome: FtsZ, SepF, FtsW, FtsX, FtsI, and FtsQ (27), suggesting a role in divisome recruitment reminiscent of that of FtsA (e.g., 32). It remains to be studied whether ZipN has any influence on FtsZ polymerization. *Anabaena* SepF interacts with FtsZ, ZipN, and FtsX, and in contrast to the general effect of SepF promoting FtsZ bundling (e.g., 33), in *Anabaena,* it limits FtsZ polymerization, influencing the morphology of the FtsZ polymers (30). Notably, in *Anabaena*, SepF interaction with FtsZ requires the N-terminal peptide of the latter (24). Perhaps, SepF is required *in vivo* at precise levels to modulate an intrinsic excessive capacity of FtsZ for self-interactions.

### Formation of the intercellular septa in the filament

As mentioned above, in the *Anabaena* filament, the neighboring cells are separated by an engrossed peptidoglycan wall. This peptidoglycan and the cytoplasmic membranes of both cells are traversed by the multiprotein complexes of the septal junctions that connect their cytoplasm, allowing intercellular molecular exchange between them (Fig. 1). The nanopores through which the septal junction complexes traverse the peptidoglycan are drilled by peptidoglycan-processing enzymes, AmiC-like amidases, once the septal peptidoglycan mesh has closed at late cell division steps (16).

**Fig 4:**
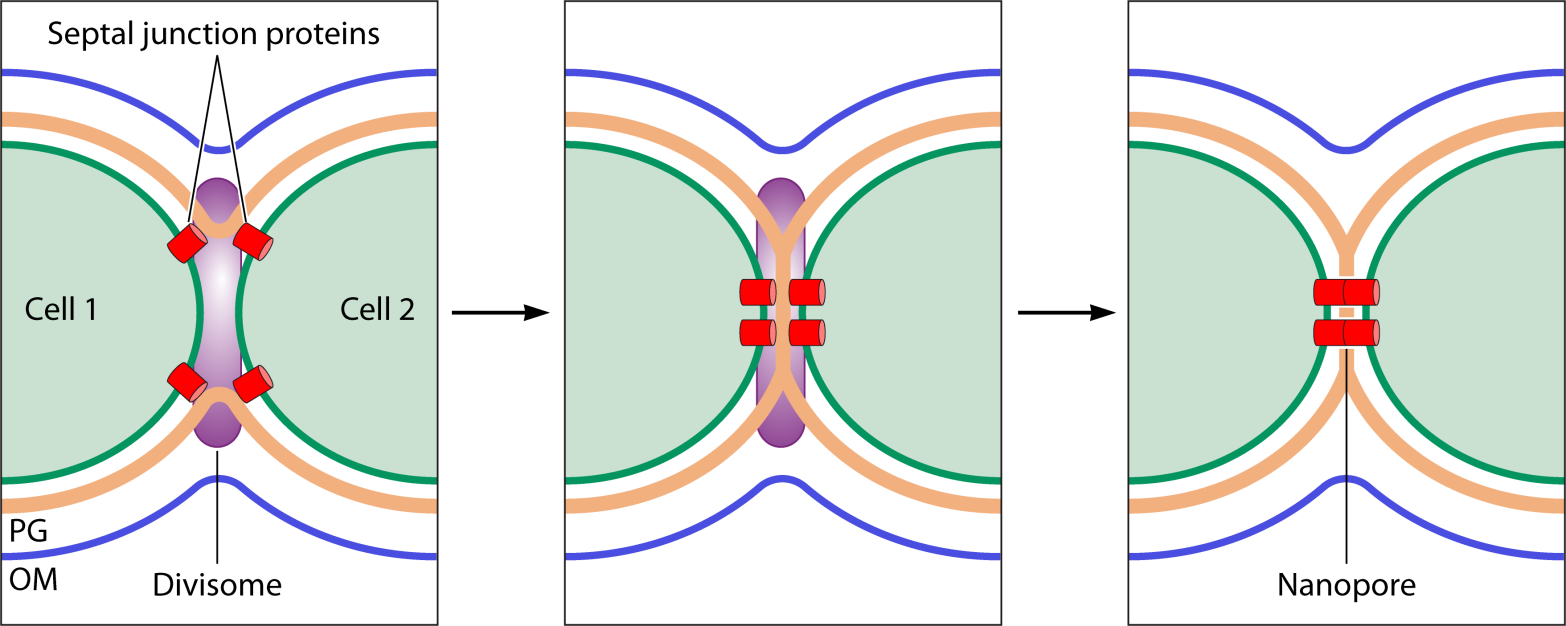
A model of the last steps of cell division in *Anabaena* depicting recruitment of septal junction proteins (red) by interaction with the divisome (purple), septal peptidoglycan closure and nanopore drilling, and localization of septal junctions.

A remarkable aspect that relates cell division to intercellular communication is that several components of the septal junction complexes have been found to interact with the divisome during cell division, which represents a way for their localization in the new septa of the daughter cells, where septal junctions remain after the divisome is dismantled (Fig. 4). Septal junction component localization to the divisome has been observed making use of protein fusions to the green fluorescent protein (GFP) and, in some cases, by immunolocalization (e.g., 34). Moreover, in the case of SepJ, it has been shown that its septal localization requires a functional divisome, being delocalized in strains that conditionally down-express *ftsZ* (35) or *zipN* (27). In addition, interactions between septal junction and divisome components have been studied by BACTH assays and by co-immunoprecipitation. Divisome-localized septal proteins include SepJ, which interacts with ZipN, SepF, FtsQ, FtsW, and FtsI (27, 30, 35); FraC and FraD (36); SepI, which interacts with ZipN, SepF, and FtsI (17); and SepT, which interacts with ZipN, SepF, and FtsW (19).

## CONNECTIONS BETWEEN CELL GROWTH AND DIVISION IN *ANABAENA*

**Fig 5:**
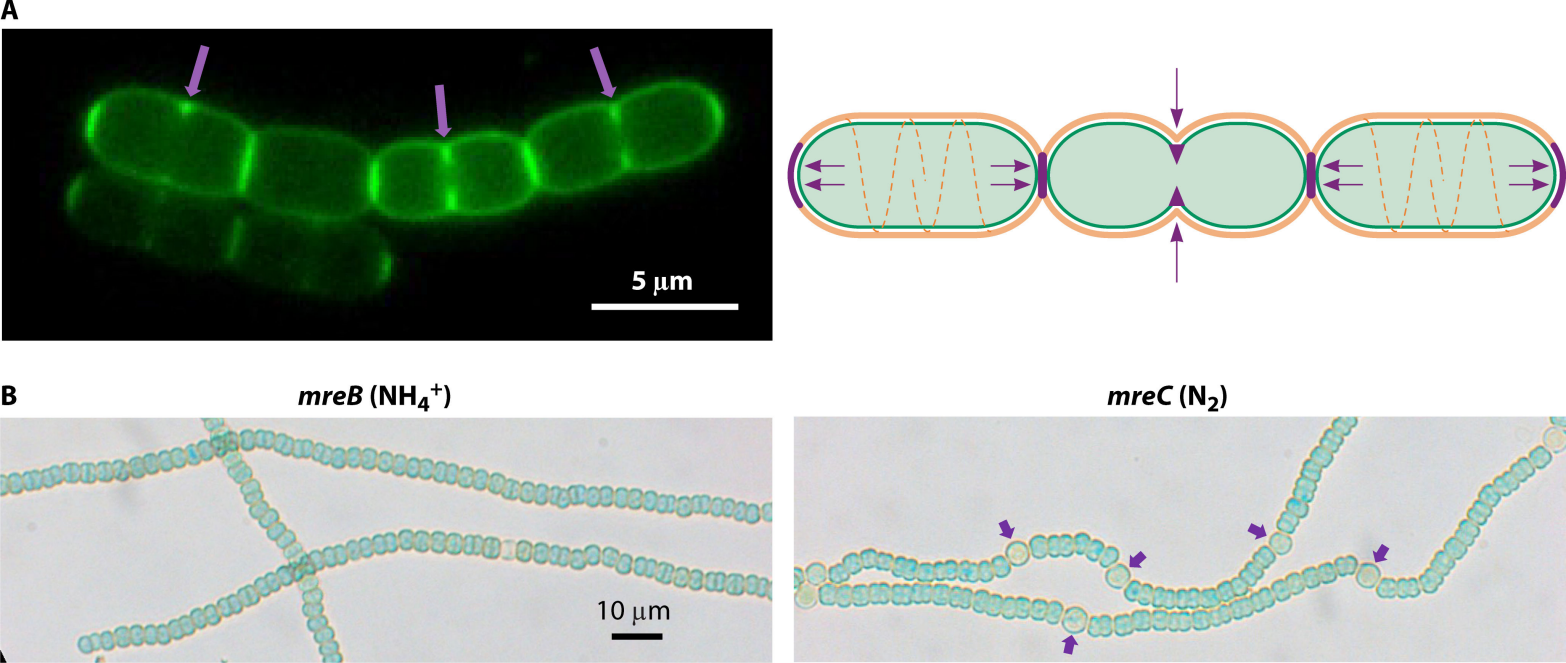
(**A**) Modes of peptidoglycan growth in *Anabaena*. Confocal microscopy image of a filament grown with ammonium and labeled with fluorescent vancomycin (Van-FL), which is incorporated in the sites of peptidoglycan addition. Arrows point to labeling at the divisome localization (left part). On the right, schematic of peptidoglycan incorporation: at the cell periphery (brown), at the divisome, and at the mature intercellular septa (purple). (**B**) Micrographs of filaments of strains CSCV1 (*mreB*) incubated with ammonium and CSCV4 (*mreC*) incubated in the absence of combined nitrogen (diazotrophic conditions). Some heterocysts are indicated with purple arrows in the latter. Magnification is the same for both micrographs (compare to filaments of the WT in Fig. 1). Figure 5B is adapted from Reference 43.

Model rod-shaped bacteria present two main modes of peptidoglycan growth: peripheral (lateral) growth to elongate the cells between cell division events, which is directed by the protein complex of the elongasome, and divisional growth, to synthesize the new poles of the daughter cells during cell division, which is directed by the divisome (37–39). In addition to these modes of growth, *Anabaena* exhibits a continuous activity of peptidoglycan incorporation in the mature septa between consecutive cells along the filament, an activity that persists after the divisome has been dismantled (40) (Fig. 5A). This pattern is in contrast to that in *E. coli* (41) or *B. subtilis* (42), in which the cell poles are inert for peptidoglycan incorporation during most of the cell cycle. It has been considered that in the mature septa of *Anabaena*, a continuous incorporation of peptidoglycan could contribute to the maintenance of intercellular structures, including the cell-to-cell communication arrays of septal junctions (40).

**Fig 6:**
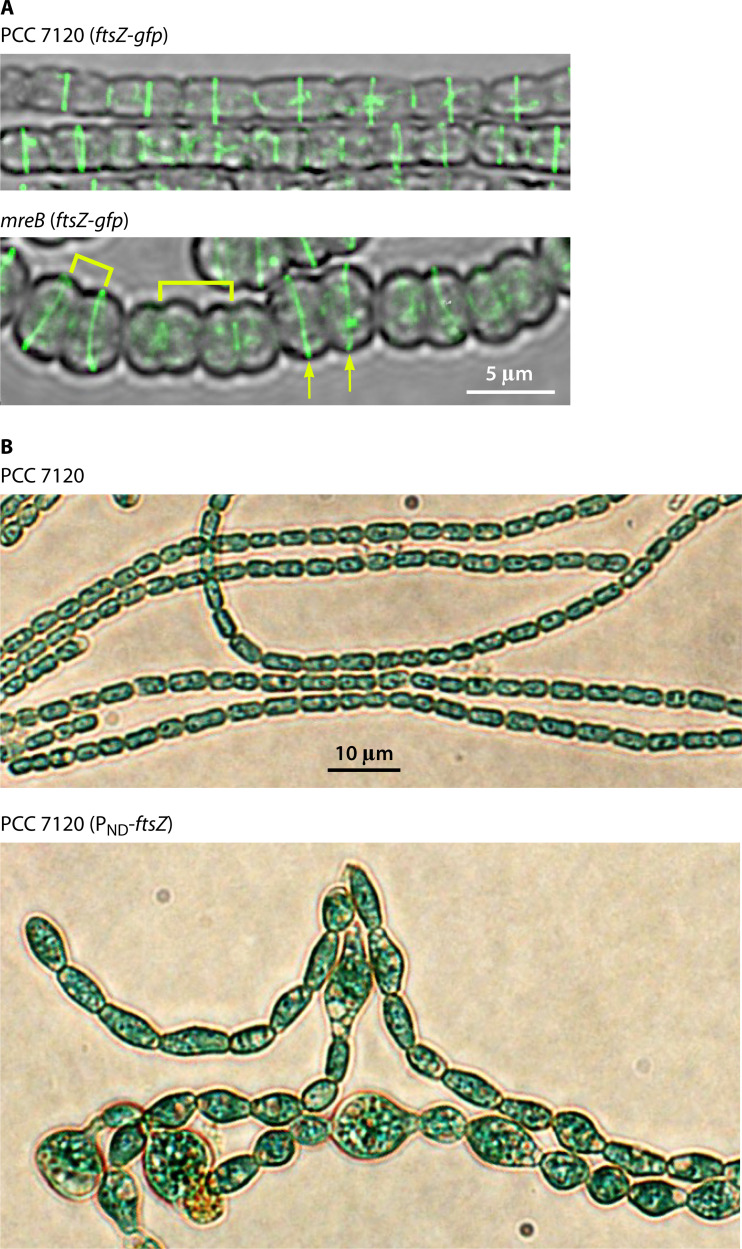
(**A**) Confocal microscopy images of ammonium-grown filaments of strains CSSC19 and CSCV20, expressing an FtsZ-GFP fusion in a wild-type or an *mreB* mutant background, respectively (see Reference 40). In the latter, some tilted FtsZ rings in consecutive cells are indicated with arrows, and brackets signal uneven distances between consecutive rings. Magnification is the same for both micrographs. (**B**) Light micrographs of ammonium-incubated filaments of strains PCC 7120 and CSFR18, which express the *ftsZ* gene from the N-regulated promoter P_ND_, determining low expression levels in the presence of ammonium (see Reference 24). Magnification is the same for both micrographs.

The *Anabaena* genome encodes homologs of the elongasome components MreB, MreC, and MreD, which in model rod-shaped bacteria contribute to localization and regulation of this complex (37–39). Consistent with an involvement in lateral peptidoglycan growth, *Anabaena* MreB, MreC, and MreD are localized to the cell periphery, but also distinctly to the divisome during all stages of cell division and to the mature intercellular septa throughout the filament (40). Mutants lacking any of these Mre factors are viable under conditions of moderate or slow growth, although exhibiting conspicuous morphological alterations. These alterations include a large increase, and variability, in cell size; inversion of the cell axes so that the axis perpendicular to the filament becomes longer than the one parallel to the filament; and distortions in the filament linearity (Fig. 5B). In addition, the septal peptidoglycan incorporation bands between consecutive cells appear wider and stronger in the *mre* mutants than in the wild type (40). Indeed, the *mre* mutants present alterations in the disposition of the cell division ring, showing instances of consecutive rings forming angles or being separated by uneven distances, in contrast to the wild type that shows consecutive parallel and equidistant rings (40) (Fig. 6A). These alterations in Z-ring geometry correlate with alterations in the plane of septal peptidoglycan incorporation (visualized with fluorescent vancomycin), which results in the observed aberrations of filament morphology (40). Therefore, in *Anabaena*, besides their canonical role in cell elongation, the Mre factors influence cell division, being required not only for lateral growth but also for the correct geometry and dimensions of the divisional and septal peptidoglycan.

Conversely, in *Anabaena*, downregulation of the genes encoding the essential cell division proteins FtsZ (35) or ZipN (27) affects not only cell division but also the elongasome activity. Thus, under these conditions, giant and strongly deformed bulged or rounded cells are observed (Fig. 6B) together with abundant cell lysis and impaired peptidoglycan incorporation at the cell periphery and the mature septa, which collectively indicate an incapacity for cell elongation without distortion of the cell width (40). This contrasts with the effects of FtsZ depletion in *E. coli* (44) or *B. subtilis* (42) that result in the formation of very long cells, indicative of continued cell elongation. Thus, in *Anabaena,* an intact divisome is also required for the proper activity of the elongasome.

Finally, consistent with functional relations between the elongasome and the divisome in *Anabaena*, direct interactions between components of both complexes (MreB interacts with FtsQ; MreD interacts with FtsW and FtsI) have been observed by BACTH assays (40).

## RESTRICTION OF CELL DIVISION DURING HETEROCYST DIFFERENTIATION

The process of heterocyst differentiation is commonly divided into three temporal phases: (i) initiation, which includes perception of nitrogen deficiency and early gene regulation responses that trigger the differentiation of individual cells and set the initial spatial differentiation pattern; (ii) commitment, which is associated, or at least coincident in time, with loss of the capacity for cell division; and (iii) morphogenesis of the differentiating cells, including specific envelope deposition and expression of genes encoding the nitrogen fixation machinery. A sequence of regulatory events affecting cell division genes and proteins has been characterized. Expression of the *ftsZ* gene in the differentiating cells takes place at levels similar to those observed in the non-differentiating, vegetative cells, becoming undetectable only in mature heterocysts after the differentiation process has been completed. However, in differentiating cells, the FtsZ protein localized in Z-rings is detectable only at early stages and becomes undetectable before differentiation is completed (45, 46). Regarding ZipN, it becomes undetectable in the differentiating cells very early (46), which is also the case for the SepF protein and *sepF* gene expression (30). Thus, in the differentiating cells, ZipN and SepF are early downregulated, preventing the formation of functional division rings. The observation that the expression of the *ftsZ* gene is maintained longer than Z-rings suggests that for some time, the FtsZ protein can persist not associated with the rings. It has been proposed that the levels of free FtsZ could represent a checkpoint in the regulation of the progress of heterocyst differentiation (47). Regarding mechanisms of inhibition of cell division, a number of proteins have been involved in this process, some of which are presented below.

### The role of PatA

PatA is a 379-amino acid protein with a putative phospho-acceptor domain (48). The *patA* gene is expressed at very low levels in cells growing with combined nitrogen, and its transcription is transiently activated in the differentiating cells at intermediate stages of differentiation. Inactivation of *patA* leads to an increase in cell size and precludes the differentiation of intercalary heterocysts in the filament (46, 48). Several observations relate PatA to the regulation of cell division: (i) when overexpressed, a PatA-GFP reporter is localized to midcell rings (49); (ii) *patA* inactivation leads to an increase in FtsZ-GFP engaged in midcell rings in the presence of nitrate (46); and (iii) PatA can interact with the essential Z-ring components ZipN and SepF (46). It has been proposed that in the differentiating cells, PatA is an essential negative regulator of the formation of the Z-ring. Besides that, PatA has been detected localized at the heterocyst poles and to interact with the septal protein SepJ, suggesting a role of PatA in the intercellular transfer of morphogens affecting the pattern of heterocyst differentiation along the filament (46).

### The role of PatD

The *patD* gene encodes a 119-amino acid protein with no recognizable domain. It is transiently upregulated late in heterocysts, and its inactivation increases the heterocyst frequency and the frequency of FtsZ rings in vegetative cells, whereas its overexpression leads to increased cell size and aberrant cell division (47, 50). PatD interacts with FtsZ and ZipN and interferes with FtsZ polymerization *in vitro*, so that in its presence, FtsZ forms aberrant thick straight bundles that differ from the toroidal aggregates formed by FtsZ alone (47). Thus, PatD represents a negative effector of FtsZ polymerization and cell division that, in the wild type, would inhibit Z-ring formation principally in the heterocysts. Accordingly, in contrast to the wild type, *patD* mutants retain some activity of midcell peptidoglycan incorporation in the heterocysts (47). In addition, it has been proposed that PatD would limit the amount of free FtsZ and its putative positive effect on the completion of differentiation (47).

### The role of ThyD

The *Anabaena* All2390 protein has been considered to represent a putative homolog of the protein SulA, a component of the SOS response of *E. coli* that interacts with FtsZ and inhibits cell division (51). However, the sequence similarity of All2390 to canonical SulA of model bacteria is insignificant, and we have renamed it ThyD (52). ThyD is localized in thylakoid membranes, being involved in the acclimation of the cells to conditions of high light intensity. Both inactivation and overexpression of *thyD* lead to instances of increased cell size, and ThyD interferes with the *in vitro* polymerization of FtsZ, leading to the formation of thinner and deformed FtsZ polymers in comparison to those formed by FtsZ alone. Thus, ThyD appears to act as a cell division effector. In addition, upon N-stepdown, the expression of *thyD* becomes higher in heterocysts than in vegetative cells, and whereas *thyD* inactivation decreases heterocyst frequency, its overexpression increases it and retards Z-ring dismantlement in the differentiating cells (52). Finally, ThyD interacts with PatD, suggesting that ThyD might represent an antagonist of the negative effector PatD during heterocyst differentiation (52).

### The role of HetZ and PatU3

In *Anabaena*, the gene cluster *hetZ-patU5-patU3* is expressed mainly in differentiating cells and heterocysts. Whereas inactivation of *hetZ* abolishes differentiation, inactivation of *patU3* produces multiple contiguous heterocysts in the absence of combined nitrogen. Inactivation of *patU5* has no noticeable effect on differentiation (53). HetZ positively modulates the expression of genes required for differentiation, such as *hetR*, which encodes the essential transcription factor HetR (54). PatU3 has a dual regulatory role: (i) it inhibits heterocyst differentiation by interaction with HetZ, limiting HetZ availability for gene activation (54, 55); (ii) it limits cell division at the step of septal peptidoglycan synthesis (55, 56) and influences the geometry of the cell division ring (57). PatU3 interacts with the divisome component ZipS (56), which, although poorly studied in *Anabaena*, has been related to peptidoglycan synthesis in the unicellular cyanobacterium *Synechococcus* sp. strain PCC 7942 (58).

### The role of HetF

The *hetF* gene is expressed in vegetative cells and downregulated in mature heterocysts (59). The heterocyst-specific RNA NsiR1.1, which is transcribed antisense to the 5′ UTR of *hetF*, could contribute to downregulation of the expression of *hetF* (60). HetF is essential for heterocyst differentiation (61) in the transition from commitment to morphogenesis (62) and for survival under conditions of high light intensity, specifically in the presence of nitrate (59). HetF localizes to the divisome coincident with the places of midcell and septal peptidoglycan incorporation and interacts with FtsI, a peptidoglycan synthase of the divisome. HetF is required for septal peptidoglycan synthesis under high light with nitrate (59), although *hetF* inactivation affects cell division under any condition (55, 62). HetF shows a protease activity that can cleave PatU3, thus limiting the amount of this negative regulator (55).

**Fig 7:**
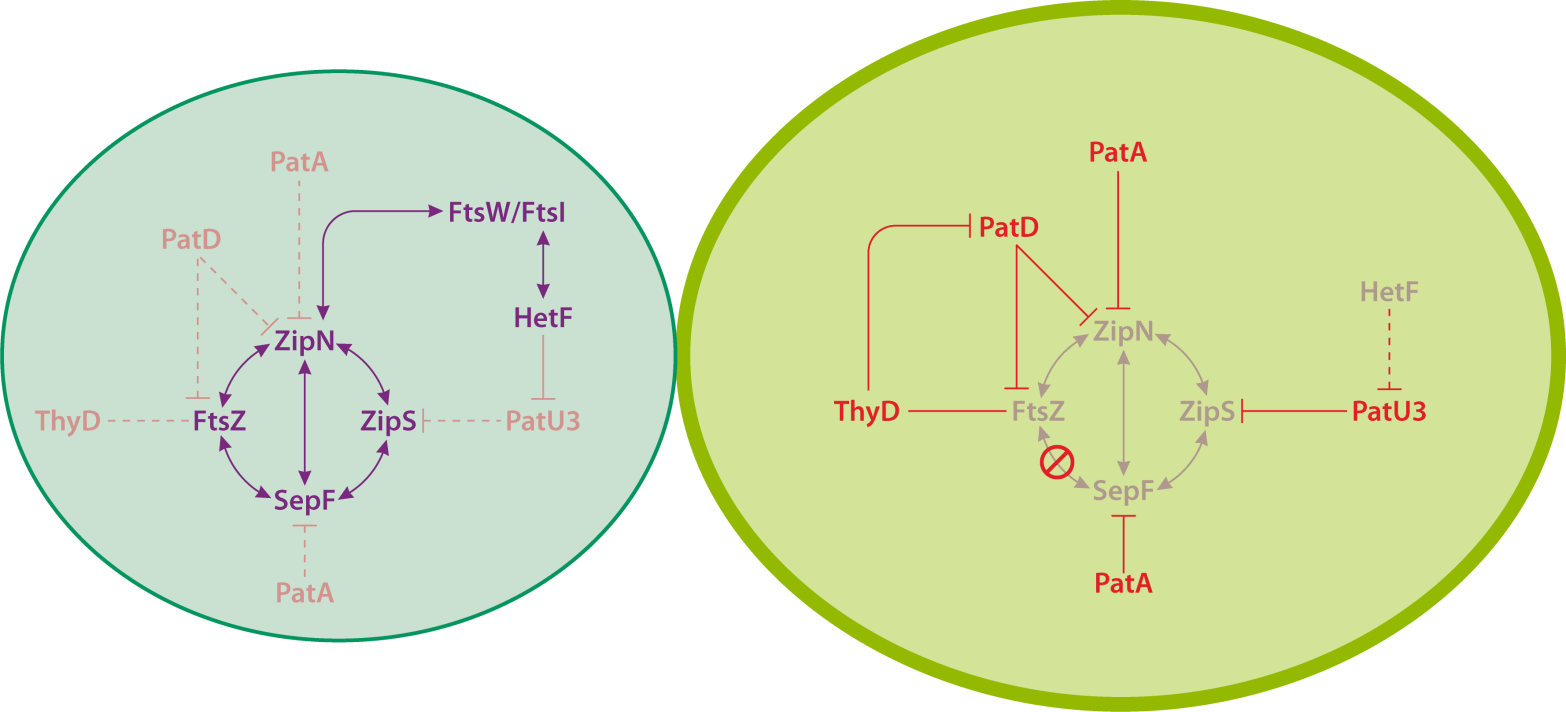
Integrated model of the multifactorial circuit of proteins contributing to cell division inhibition during heterocyst differentiation. Cell division proteins are represented in purple. Proteins that interact with cell division proteins and influence cell division and heterocyst differentiation are indicated in red. Light colors represent low expression levels, and dark colors represent high expression levels. Red lines indicate protein-protein regulation (low-level regulation indicated by dashed red lines). PatA and HetF are required for, and ThyD favors, differentiation. PatD and PatU3 limit differentiation. PatA and PatD limit the formation of the Z-ring, HetF favors septal PG growth, and PatU3 influences the geometry of division. SepF downregulation in differentiating cells prevents FtsZ polymerization (indicated by a red restricted signal). HetF could degrade PatU3. A vegetative cell is represented to the left, and a cell differentiating into a heterocyst to the right.

Figure 7 presents a model integrating the role of the above-described proteins in the regulation of cell division during heterocyst differentiation. In summary, PatA and PatD would inhibit Z-ring formation by interfering with the essential components FtsZ, ZipN, or SepF, and PatD might additionally limit the levels of free FtsZ required to inform for the progression of differentiation. ThyD influences FtsZ polymerization and would represent an inhibitor of PatD, limiting its influence on both the Z-ring and free FtsZ. PatU3 would inhibit the further activity of the divisome by interacting with ZipS. Finally, because the limitation of the PatU3 levels by HetF appears to be required, and because the *hetF* gene is preferentially expressed in the vegetative cells, differentiation could be influenced by the activity of HetF in these cells.

## CONCLUSIONS AND PERSPECTIVES

Filamentous cyanobacteria of the order Nostocales show a multicellular behavior that is unique in the bacterial kingdom. Key to this organization is the structure of the cyanobacterial filament of communicating cells, which is propagated by the concourse of cell division proteins that are specific or have specific features with regard to those found in model unicellular bacteria. In addition, proteins related to cell division participate in the process of differentiation of heterocysts. Intercellular structures for cell-to-cell communication include septal junction protein complexes that are localized by interaction with the divisome during cell division. In setting the structure of the *Anabaena* filament, specific features are also found in relation to peptidoglycan growth, which in *Anabaena* relies on interdependent activities of the elongasome and the divisome complexes and persists in the mature intercellular septa. Worth of future study are details of septal peptidoglycan construction, including nanopore drilling influenced by septal junction proteins, with the concourse of peptidoglycan-processing enzymes acting by a mechanism that largely remains unresolved.

Inhibition of cell division during heterocyst differentiation sets the point of commitment (i.e., of no return to the vegetative cell state). Genes *ftsZ*, *zipN*, and *sepF*, encoding Z-ring components, are downregulated by a mechanism that has not been investigated. Besides, the levels of the corresponding proteins and their incorporation into functional division rings are negatively regulated. A compelling issue for future work is to gain an integrated knowledge of the mechanism of action of the multiple elements participating in the dismantlement of the Z-ring during heterocyst differentiation. Finally, it is worth stressing that various proteins that are induced during differentiation and negatively regulate cell division in differentiating cells have been shown to exert some effect limiting cell division also in the vegetative cells, an intriguing issue that deserves future investigation (Fig. 7).
